# What is cholera? A preliminary study on caretakers’ knowledge in Bangladesh

**DOI:** 10.1186/s41043-016-0040-6

**Published:** 2016-02-09

**Authors:** Charlotte C. Tamason, Suhella M. Tulsiani, A. K. Siddique, Bilqis A. Hoque, Peter K. Mackie Jensen

**Affiliations:** 1 Department of Public Health, Global Health Section, University of Copenhagen, Øster Farimagsgade 5, Building 9, 1353 Copenhagen, Denmark; 2 Copenhagen Centre for Disaster Research, University of Copenhagen, Øster Farimagsgade 5, Building, 1353 Copenhagen, Denmark; 3 International Centre for Diarrhoeal Disease Research, GPO Box 128, Mohakhali, Dhaka, 1000 Bangladesh; 4 Environment and Population Research Centre, House# 292, Road# 19/B, New DOHS Mohakhali, Dhaka, 1206 Bangladesh; 5 Department of Civil and Environmental Engineering, Uttara University, House# 4, Road# 15, Uttara, Dhaka, 1230 Bangladesh

**Keywords:** Bangladesh, Cholera, Diarrhea, Qualitative research, Social stigma

## Abstract

**Background:**

Cholera has afflicted the Indian sub-continent for centuries, predominantly in West Bengal and modern-day Bangladesh. This preliminary study aims to understand the current level of knowledge of cholera in female Bangladeshi caretakers, which is important in the outcome of the disease and its spread. A pilot study was conducted among 85 women in Bangladesh using qualitative questionnaires to explore the ability of female caretakers in identifying cholera and its transmission.

**Findings:**

The survey revealed that though all the female caretakers were aware of the term “cholera,” nearly a third of the respondents did not associate diarrhea with cholera or mentioned symptoms that could not be caused by cholera (29 %). Approximately half of the respondents associated water with the cause of cholera (56 %) and only 8 % associated cholera with sanitation or hygiene. Shame and stigma (54 %) were more commonly described than death (47 %) as negative effects of cholera.

**Conclusions:**

The results from this study are suggestive of a need for reformulation of cholera and diarrhea communication. Messaging should be based on signs of dehydration, foregoing the use of medical terminology.

## Findings

### Rationale

Household caretakers are the gatekeepers to seeking and providing treatment for cholera within a household. Their ability to identify an episode of diarrhea in a family member as cholera is paramount to timely disease management. This survey focuses on understanding a primary caretaker’s knowledge and perception of cholera in Bangladesh and aims to present the importance of communicating cholera and diarrhea prevention and treatment messages focused on dehydration and danger signs as opposed to case definitions.

### Materials and methods

This study consisted of two study sites: Dhaka and Shyamnagar, representing urban and rural Bangladesh, respectively.

The study sites in Dhaka included in this study were Kurail, Sattala, Kallanpur, Basila, and Rupnagar slum areas.

Shyamnagar is an Upazilla (sub-district), located in the southwest Bangladesh. The survey was conducted in Shyamnagar, Sathkira, Munshingonj, BuriGoalini, Ramzan-Nagor, Atolia, and Padmapukur Unions.

The study population included 85 women caretakers (43 from Shyamnagar and 42 from Dhaka) between the ages of 18 and 55 years with household caretaker responsibilities. A caretaker was defined as the person that is primarily in charge of caring for children, cooking, cleaning, and collecting water or oversees these activities for the household.

Data for this study were collected through questionnaires, consisting of 26 open-ended questions, from January to February 2011. Households were selected randomly by starting in the center of a slum or union, throwing a stick in the air, and walking in the direction the stick pointed, with a minimum of 5 min walking distance between each household surveyed. Interviews lasted an average of 28 min each.

After collecting demographic data, the study participants were asked, “What is cholera?” as well as cholera’s cause, how it was spread, and about its main negative effects and social effects. The responses were coded numerically in Bengali and then translated to English. Multiple answers and different wordings were given separate numerical corresponding codes. For example, a certain response was given three numerical codes because a woman described cholera as (1) “A serious disease,” that included (2) “Loose stools,” and (3) “The stool had a very bad odor.”

Cholera for this study is defined as acute watery diarrhea with or without vomiting, as per World Health Organization (WHO) guidelines [1]. Responses were then classified as being correct, partially correct, or incorrect. Analyses were performed using SPSS.

### Ethics, consent, and permissions

No biological samples were collected, and no individual data were shared. The study was conducted through collaboration between the Environment and Population Research Centre (EPRC) and the University of Copenhagen under the project “The Impacts of Climate Change on Water, Sanitation and Hygiene and its Influence on Livelihood and thereby Human Security in Bangladesh,” which was approved by the EPRC executive committee and the NGO Bureau of Bangladesh in 2011. Women were informed about the aims of the study, the involved institutions, and that their participation was voluntary during a verbal consenting process.

### Results

Demographic information for Dhaka Shyamnagar were similar with the exception of literacy, which was lower in the rural setting (22 %) than in Dhaka (44 %). Education levels ranged from illiterate and 0 years of formal education to fazel (Arabic equivalent to a bachelor’s degree). Household income ranged from approximately $135 to $2700 USD/annum, and 95 % of the families lived on less than $1 per person per day. Most respondents were married (88 %). The majority were housewives (66 %), followed by day laborers (27 %), and others (7 %).

No significant differences were recorded between urban and rural knowledge on cholera (odds ratio = 0.86 [95 % confidence interval (CI): 0.34, 2.20]). Therefore, results are presented for the population as one.

The women of the study population were first asked, “What is cholera?” (Table 1). None of the women responded that they did not know what cholera was. Zero women described cholera as acute or watery diarrhea, and eight women described it as *ola-utah*—a term that is historically thought to denote cholera [2]. During most interviews, the words “diarrhea” and “cholera” were seemingly used as interchangeable terms for a severe sickness (Table 1).

**Table 1:** List of terms given to describe cholera symptoms by the study population (*N* = 85)

	Symptoms given	*N* = 85 people (% of total)
Incorrectly described cholera or failed to mention loose or frequent stools	Blood in stool, frequent blood in stool, chest pains, twisting of hands and feet, blood in mouth, bleeding from the belly, dysentery, very bad smell of feces	25 (29 %)
Partially correctly described cholera	Loose or frequent stools AND any of the following: vomiting, frequent vomiting, dry eyes, dry mouth, headache, stomach pain, fever, belly pain, shaking/convulsions, a serious disease, a water-borne disease	60 (71 %)
Correctly described cholera	Acute watery diarrhea/stools with or without vomiting	(0 %)

Next, the respondents were asked the cause of the cholera (Table 2). Nearly all (84 %) of the women in the study responded in some form that human behavior was the cause of cholera.

**Table 2:** Themes identified in the study population’s responses to the question, “What causes cholera?” (*N* = 85). Multiple answers possible

Themes in responses	Number of caretakers that mentioned this theme (*N* = 85) (%)
Water	48 (56 %)
Food	22 (26 %)
Flies and mosquitoes	9 (11 %)
Hygiene	7 (8 %)
Sanitation	5 (7 %)
Others	4 (5 %)

The women were then asked how they believed cholera was spread. A vast majority (97 %) described at least one potential or known route of cholera transmission either through food, water, hygiene, or sanitation.

**Fig. 1:**
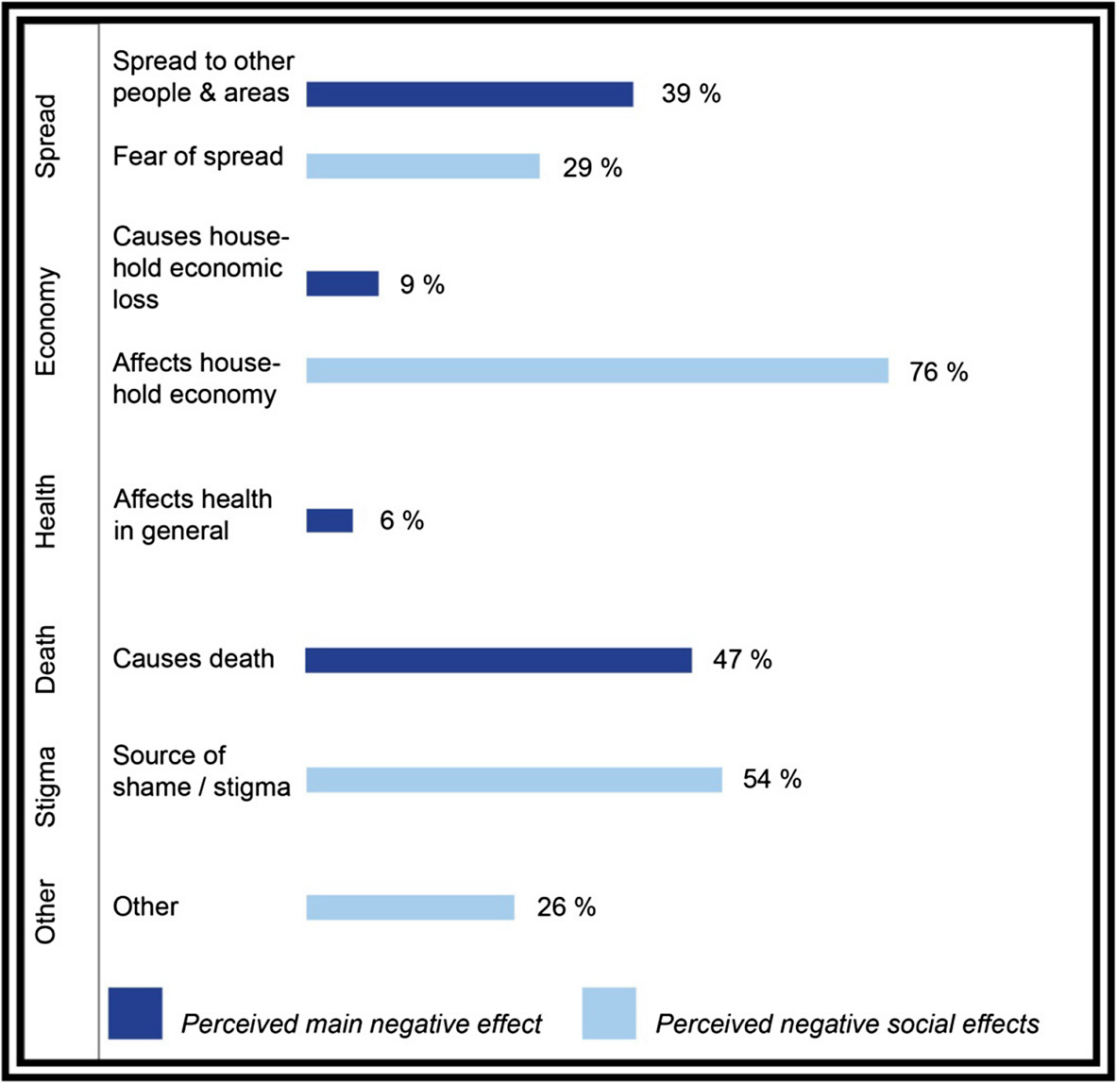
The main negative effect and social effects of cholera given by the study populations (*N* = 85). Multiple responses possible

When asked about cholera’s primary negative effect, all responses fit within four categories: spread, economic loss, general health, and death (Fig. 1).

The final question on perception addressed social effects of cholera (Fig. 1). Women provided many more detailed responses for this question than other questions. The most common theme of answers to this question was economy-related (e.g., inability to work) (76 %). “Other” answers (26 %) included issues in cleanliness, cooking, esthetics, and some seemingly unrelated responses such as “cannot work due to coughing.”

### Discussion

The Sanskrit word *visuchika* accurately describes cholera symptoms and is thought to be used to identify cholera in 400 B.C. [2, 3]. *Ola-utah*—vomiting and purging—is another term believed to denote cholera and has been used to describe the disease by the general population of Bangladesh during recent centuries [2]. With the spread of conventional medicine, *ola-utah* was replaced by the Latin word, cholera. In 1978, the WHO’s global Diarrhoeal Diseases Control Programme (CDD) was created to improve knowledge on and promote diarrhea prevention [4], and cholera was eventually reclassified in Bangladesh as “acute watery diarrhea.”

From 2010 to 2012, *Vibrio cholerae*, the causative agent of cholera, were extracted from 16 % of diarrhea cases from hospitals in different sites in Bangladesh [5]. We observe that women in our study recognized the word cholera but failed to mention specific descriptive characteristics associated with cholera, e.g., “watery stools” [6]. We argue that this may be indicative of a general trend of a mismatch between the term cholera and identification of symptoms at the household level. A recent study in urban Dhaka found that only 23 % of people could recognize cholera as acute watery diarrhea [7], which differs from this pilot study in which no respondents mentioned watery consistency or duration of diarrheal episode. This lack of knowledge also differs from perception studies in endemic areas in Africa where most respondents can reportedly identify cholera [8, 9].

Limitations of this study include the relatively taboo subject of diarrhea/loose stools that may have prevented some women from describing cholera symptoms in more detail. However, the data collector was experienced and asked probing questions to elicit more descriptive responses if the respondent appeared shy or embarrassed. It is assumed that some information was lost in translation. Our household selection method may have led to more households being selected from the center of a slum/union than the peripheries; however, socio-economic status was varied throughout the communities, so it should not have affected results.

Our study also revealed that women had limited knowledge on the source of cholera and its transmission, as most of them provided only one or two routes through which the disease could be spread. This implies that they are not aware of important measures of prevention. Furthermore, respondents readily gave many examples of stigma of the disease. Studies on HIV, mental health, and sexually transmitted diseases [10–12] demonstrate the negative consequences that stigma can have on care- and treatment-seeking rates, which may very well be happening, although perhaps to a lesser extent, in Bangladesh with cholera [12].

### Conclusions

This study suggests that reliance on household case definitions of cholera in Bangladesh may not be possible and perhaps counterproductive. Vigorous efforts should be made to educate at-risk populations, most importantly female caretakers, about the signs of dehydration, means of cholera prevention, and the importance of seeking treatment for severe dehydration [13]. Communication for prevention and treatment of cholera should be formulated based on signs of dehydration, foregoing the use of medical terminology. Furthermore, future research in Bangladesh that depends on self-reporting of cholera or diarrhea should work to develop case-definition terminology that does not incorporate the words “diarrhea” or “cholera,” even though the terms are locally recognized.
